# How to Classify and Define Pituitary Tumors: Recent Advances and Current Controversies

**DOI:** 10.3389/fendo.2021.604644

**Published:** 2021-03-17

**Authors:** Congxin Dai, Jun Kang, Xiaohai Liu, Yong Yao, Haijun Wang, Renzhi Wang

**Affiliations:** ^1^Department of Neurosurgery, Beijing Tongren Hospital, Capital Medical University, Beijing, China; ^2^Department of Neurosurgery, Xuanwu Hospital Capital Medical University, Beijing, China; ^3^Department of Neurosurgery, Peking Union Medical College Hospital, Chinese Academy of Medical Sciences and Peking Union Medical College, Beijing, China; ^4^China Pituitary Adenoma Specialist Council, Beijing, China; ^5^Department of Neurosurgery and Pituitary Tumor Center, The First Affiliated Hospital of Sun Yat-sen University, Guangzhou, China

**Keywords:** pituitary tumors, definition, classification, management, China Pituitary Adenoma Specialist Council

## Abstract

Pituitary tumors are very complex and heterogeneous and have a very wide range of proliferative and aggressive behaviors, and how to define and classify these tumors remains controversial. This review summarizes the epidemiology and progress in the classification and definition of pituitary tumors, as well as controversial issues. Based on the results of radiologic and autopsy studies, the prevalence of pituitary tumors has recently increased significantly. However, the majority of pituitary tumors are incidentally discovered and asymptomatic, and such tumors are called pituitary incidentalomas. Most of these incidentalomas do not induce symptoms, remain stable in size, and do not need treatment. The recent revised classification strategies mainly depend on immunohistochemistry (IHC) to detect pituitary hormones and pituitary transcription factors; therefore, the accuracy of diagnosing pituitary tumors has improved. Although new classification strategies and definitions for pituitary tumors have been presented, there are still some controversies. The term pituitary neuroendocrine tumor (PitNET) was proposed by the International Pituitary Pathology Club, and this terminology can encompass the unpredictable malignant behavior seen in the subset of aggressive pituitary adenomas (PAs). However, some endocrinologists who oppose this change in terminology have argued that the use of tumor in the terminology is misleading, as it gives PAs a harmful connotation when the majority are not aggressive. Such terminology may add new ambiguity to the origin of PAs and unnecessary anxiety and frustration for the majority of patients with benign PAs. The classification of aggressive PAs mainly relies on subjective judgment of clinical behavior and lacks objective biomarkers and unified diagnostic criteria. However, the term “refractory” could more accurately represent the characteristics of these tumors, including their clinical behaviors, radiological features, and pathologic characteristics. Moreover, the diagnostic criteria for refractory PAs are stricter, more objective, and more accurate than those for aggressive PAs. Early identification of patients with these tumors through recognition and increased awareness of the definition of refractory PAs will encourage the early use of aggressive therapeutic strategies.

## Introduction

Pituitary tumors arise from anterior pituitary cells, most of which are generally benign tumors and are classified as pituitary adenomas (PAs); only 0.1–0.2% of tumors present craniospinal or systemic metastasis, and these tumors are known as pituitary carcinomas (PCs) (1). Although the classification of pituitary tumors has been modified significantly in recent years, there are still some controversies (2, 3). The China Pituitary Adenoma Specialist Council (CPASC) established in 2012 includes experienced neurosurgeons, endocrinologists, pathologists, radiologists, and scientists. To introduce newer concepts into the classification and definition of pituitary tumors, the CPASC has summarized the recent advances and controversial issues in the classification and definition of pituitary tumors. This short review presents the epidemiology, new classification strategies, and new definitions of pituitary tumors, as well as controversial issues.

## Prevalence and Incidence of Pituitary Tumors

Pituitary tumors account for approximately 10–15% of all intracranial neoplasms and are the second most common primary brain tumors in humans (4). A meta-analysis of autopsy and imaging evaluation studies found an overall estimated prevalence of PAs of 16.7% (14.4% in autopsy studies and 22.5% in radiologic studies) (5). With the wide application of magnetic resonance imaging (MRI), the prevalence of PAs has increased significantly in the last 10 years. According to the results from several cross-sectional community-based studies, the prevalence of PAs varies from 78 to 116 per 100,000 individuals (6–9). However, a majority of adenomas are incidentally discovered and asymptomatic, and such tumors are called pituitary incidentalomas. Currently, the accepted definition for a pituitary incidentaloma is an unexpected pituitary/sellar lesion discovered on an imaging study performed for an unrelated reason (10). The prevalence of pituitary incidentalomas is rather high, and a previous autopsy series revealed a mean prevalence of 10.7%, with the frequency of identified pituitary incidentalomas ranging from 1.5 to 31% (11). A retrospective analysis of 353 patients from one institution reported that pituitary incidentalomas represented 12% of all pituitary tumors (12). The majority of pituitary incidentalomas are non-functioning adenomas that do not induce symptoms and remain stable in size over long-term follow-up and therefore do not need treatment (13). Only approximately 11–13.3% of pituitary incidentalomas will increase in size and need therapeutic intervention (13, 14). Within one million people, approximately 100,000 harbor PAs; however, only 0.1% of these PAs cause clinically significant health problems (15). Among patients with PAs who experience significant clinical health issues, 68.4% of them (range, 62.2–77.3%) are female, and 47.8% (range, 41.3–56.9%) have macroadenomas at diagnosis (16). Prolactinomas are the most prevalent (32 to 66%), followed by non-functioning adenomas (NFPAs) (14 to 54%), acromegaly (8 to 16%), Cushing’s disease (2 to 6%), thyrotropin-secreting adenomas (less than 1%), and the very rare PCs (0.1 to 0.2%) (17, 18).

## Advances in the Classification and Definition of Pituitary Tumors

Pituitary tumors are divided into PAs and PCs based on whether they show cerebrospinal and/or systemic metastasis. Before the release of the 4th edition of the World Health Organization (WHO) guidelines for the classification of pituitary tumors in 2017, PAs were classified as invasive or non-invasive, typical, or atypical, and aggressive or non-aggressive PAs according to their radiological characteristics, pathologic features, and clinical behavior (19). In the 4th edition of the guidelines for the classification of pituitary tumors, the term “atypical adenoma” was abandoned due to the lack of sufficient evidence that poor prognosis can be predicted by only pathological markers (20). Another main change is the introduction of a cell lineage-based classification strategy for PAs, including classification according to pituitary adenohypophyseal cell lineage and hormones production (21). The new classification strategy for PAs has abandoned the concept of “a hormone-producing pituitary adenoma” and adopted a pituitary adenohypophyseal cell lineage-based strategy to classify PAs. In the new classification, in addition to immunohistochemistry (IHC) analysis to detect hormones, IHC to detect transcription factors is required and prioritized to classify PAs, but routine ultrastructural examination is not required for these tumors (2).

Most PAs are considered benign tumors, but approximately 10% of them have aggressive behavior and are refractory to conventional therapy. The definition of aggressive PAs has also been modified in the 4th edition of the classification of pituitary tumors. According to the recent Clinical Practice Guidelines issued by the European Society of Endocrinology, aggressive PAs are tumors that are radiologically invasive and have an unusually rapid growth rate or clinically relevant tumor growth despite optimal standard therapies (surgery, radiotherapy, and medicinal treatments) (22).

This new classification scheme also recognizes some subtypes of pituitary tumors as “high-risk Pas,” which include sparsely granulated somatotroph adenomas, lactotroph adenomas in men, silent corticotroph adenomas, Crooke’s cell adenomas, and plurinominal Pit-1-positive adenomas (23). Most of these “high-risk PAs” are invasive, large macroadenomas with a high Ki-67 proliferation index; in addition, they are usually difficult to completely resect and tend to have a high recurrence rate after surgery (24).

Compared with the previous WHO 2004 classification guidelines for pituitary tumors, the 2017 WHO classification guidelines are more practical because they enable an accurate diagnosis mostly based on the levels of pituitary hormones, pituitary transcription factors, and other commonly used markers (detected by IHC). A good correlation between the subtypes and pathological features and clinical outcomes has also been shown, which contributes to our understanding of the clinicopathological characteristics of pituitary tumors.

## Controversies in the Classification and Definition of Pituitary Tumors

Pituitary tumors are a group of tumors with complex and heterogeneous clinical features, including a very wide range of proliferative and aggressive behaviors. Some pituitary tumors are asymptomatic and remain stable in size for a long time, whereas other aggressive PAs grow rapidly and are refractory to conventional treatments. Some pituitary tumors are small in size but have severe systemic metabolic abnormalities caused by excessive secretion of pituitary hormones, whereas others are massive, invasive adenomas that cause local mass effects but do not show excessive hormone secretion. Thus, it is difficult to classify these complex pituitary tumors. Although the new classification and definition strategies for pituitary tumors have been significantly modified, there are still some controversies.

The term “pituitary adenoma” has been used to define a benign pituitary tumor for approximately a century. However, “adenoma” does not represent the malignant characteristics of aggressive PAs, such as invasion of nearby anatomical structures, rapid growth, non-response to conventional treatments, and early postoperative recurrence. More recently, a proposal to change the terminology from PA to pituitary neuroendocrine tumor (PitNET) was proposed by the International Pituitary Pathology Club (25). They argued that the term “PitNET” instead of “adenoma” permitted variability in biological behavior and aligned these tumors with the terminology used for other neuroendocrine tumors. Subsequently, the European Pituitary Pathology Group endorsed the term PitNETs and developed a practical recommendation for standardized reports and diagnostic algorithms for PitNETs (26). Trouillas and colleagues also believe that PitNETs more closely reflect the variability of pituitary tumor behavior (with invasiveness linked to a higher risk of recurrence) and that the new terminology may suggest new strategies for the early identification and management of the most aggressive tumors (27). Although a large majority of PitNETs behave as well differentiated and benign neoplasms, the terminology of PitNETs not only reflects more closely the variability of behavior of pituitary tumors, but also impact positively on clinical practice. In addition, the current classification of the anterior pituitary adenomas does not accurately reflect the clinical behavior including invasive PAs that cannot be completely resected and aggressive PAs refractory to therapy. Therefore, application of this term may open up new strategies for the early identification and management of the most aggressive forms. Therefore, they endorsed PitNET terminology as well because it could positively impact clinical practice.

However, the PitNET terminology has caused a nomenclature debate. Some endocrinologists who oppose this change in terminology argue that the use of the term tumor is misleading, as it gives PAs a negative connotation when the majority are not aggressive (only a small subset of them are aggressive adenomas), and true malignant PCs are extremely rare (28). They criticize that the PitNET term encompasses tumors with unpredictable malignant behaviors and that the PitNET terminology will add new ambiguity to the origin of PAs and bring unnecessary anxiety and frustration to patients with typical benign Pas (15). Therefore, both the adenoma and PitNET terminologies have their own pros and cons, and more research and discussion are required to verify which is more representative of the characteristics of pituitary tumors. To date, there is still no consensus or consist terminology and definitions used for these complex and heterogeneous pituitary tumors, and more accurate terminology and definitions are needed to appropriately represent the highly variable clinical features of pituitary tumors.

Most PAs are benign and non-invasive adenomas that can be cured by surgery or controlled by medicinal therapy, such as dopamine-agonist therapy for lactotroph tumors. However, approximately 35% of PAs have invaded into surrounding anatomical structures and cannot be completely resected (19). Moreover, approximately 10% of PAs with invasive characteristics and unusually rapid growth rates recur multiple times despite optimal administration of standard therapies, and such tumors are defined as clinically aggressive Pas (29). Before the release of the recent Practice Guidelines for aggressive PAs, the definitions of “aggressive tumor” varied from a large invasive pituitary tumor with rapid growth, to a tumor resistant to conventional treatment, a pituitary tumor with early recurrence despite gross-total resection, to a tumor with malignant potential without metastasis, to a localized pituitary carcinoma (30–32). Trouillas and colleagues demonstrated that aggressive PAs and pituitary carcinomas are clinically and histologically similar. Therefore, they suggested that aggressive PAs are “tumors with malignant potential without metastasis” and proposed that aggressive PAs and carcinomas may be two sides of the same coin (32). Compared with the various previous definitions for aggressive PAs, the version recently proposed by the European Society of Endocrinology is currently the most authoritative and comprehensive (22). Although the definition of aggressive PAs has been revised and modified repeatedly, there is still some controversy and confusion.

Previously, radiological investigation was used to determine the invasiveness of aggressive PAs; however, radiologically determined invasion, based on preoperative MRI, is not always consistent with intraoperatively observed invasiveness (33). The Knosp classification is based on preoperative MRI and is most widely used system to evaluate the invasiveness of Pas (34). According to this classification system, Knosp grade 0–2 tumors are non-invasive PAs, and Knosp grade 3–4 tumors are invasive PAs. However, the MRI-based Knosp grade is often unreliable for predicting intraoperative invasion in Knosp grade 1–3 microadenomas (35). A subset of MRI-based radiologically invasive tumors is found to exhibit expansive growth without cavernous sinus invasion on intraoperative inspection, whereas some radiologically non-invasive tumors are found to have cavernous sinus invasion during surgery. Therefore, radiologically determined invasion cannot accurately represent the invasiveness of aggressive PAs, and the term invasive should consider both radiological and surgical findings.

Second, there is no uniform objective standard to identify the unusually rapid tumor growth rate required in the definition of aggressive PAs. Currently, physicians determine whether the tumor is growing rapidly mainly based on their own subjective experience, which may result in different clinicians having inconsistent judgments for the same patient. Therefore, the lack of objective and unified standards for identifying an unusually rapid tumor growth rate can easily lead to inconsistent diagnoses for the same patient with an aggressive PA.

Third, the diagnosis of aggressive PAs mainly depends on subjective judgment of clinical characteristics and lacks objective biomarkers and unified diagnostic criteria. It is difficult for inexperienced physicians to diagnose aggressive PAs early and accurately. Therefore, some aggressive PAs are easily missed by inexperienced physicians due to the lack of obvious diagnostic markers and uniform objective criteria.

Therefore, the term “aggressive” is mainly applied based on clinical behaviors rather than histological markers. The definition of aggressive tumors does not accurately convey the precise clinical and pathological features of “aggressive” tumors, and there is no correlation between pathologic findings and the clinical behavior of these tumors. Furthermore, there is no biomarker used in the definition of “aggressive” that predicts the aggressiveness of these tumors. In addition, the term “aggressive” is frequently interpreted differently by individual clinicians due to the lack of objective diagnostic markers and uniform criteria. Moreover, the terms “invasive” and “aggressive” are constantly interchangeably and synonymously used in some studies. Thus, a more reasonable and comprehensive term or definition is needed to properly convey the clinical, radiological, and histological features of these aggressive PAs.

To address this situation, we proposed the term “refractory PAs” to identify invasive-aggressive PAs with a high Ki-67 index, rapid growth, early recurrence, and resistance to conventional treatments (36). This definition was derived by summarizing the clinical characteristics and pathological findings of a group of patients with refractory PAs from Peking Union Medical College Hospital (PUMCH). The diagnostic criteria for refractory PAs were also proposed as follows: 1) tumor infiltration of adjacent structures according to radiological results or intraoperative findings; 2) Ki-67 index greater than 3%, and growth velocity greater than 2% per month; 3) failure of current treatments to control tumor growth and/or hormonal hypersecretion; and 4) tumor recurrence within 6 months after surgery (37). Although the term “refractory” has caused some controversy (38), it does provide a more accurate definition and more objective diagnostic criteria for these invasive-aggressive PAs.

Instead of “aggressive,” we prefer to use the term “refractory.” Because the definition of “refractory” has several advantages over “aggressive”.

First, although both the “aggressive” and “refractory” definitions include invasive tumors, the “aggressive tumor” definition only mentions radiologically invasive tumors, which is not comprehensive or accurate. In the diagnostic criteria for “refractory tumors,” tumor infiltration into adjacent structures according to radiological results or intraoperative findings is used to determine the invasiveness of tumor, which is more comprehensive than the term “radiologically invasive tumor” used in the definition of “aggressive tumors.”

Second, Ki-67 >3%, as an objective biomarker, is used to diagnose refractory PAs, which provides an objective biomarker for these tumors. Although most studies have demonstrated that Ki-67 >3% predicts more aggressive tumor behavior, no firm consensus on the precise cutoff value has been reached (39). Therefore, the use of a cutoff value >3% for Ki-67 to distinguish refractory PAs from benign PAs is still controversial; however, such a cutoff does help physicians distinguish refractory PAs from benign PAs.

Third, a rapid tumor growth rate is mentioned in both the “aggressive” and “refractory” tumor definitions, but the “aggressive” tumor definition does not provide an objective and precise value indicating rapid growth. In the diagnostic criteria for “refractory” tumors, a growth rate >2% per month is used to define rapid growth of tumors. Although the growth rate >2% per month criterion was determined based on the summarized findings of a series of patients with refractory PAs at PUMCH, and its validity as a marker remains to be confirmed, it indeed provides an objective reference for an unusually rapid tumor growth rate. Further research is needed to explore a more acceptable cutoff value for identifying rapid growth to distinguish refractory PAs from benign PAs.

Fourth, early recurrence (6–12 months postoperatively) is another important feature of aggressiveness characterizing the behavior of aggressive/refractory PAs, whereas typical benign adenomas may develop recurrence within 5–10 years after initial treatment (40). Aggressive/refractory PAs are usually resistant to conventional treatment and recur early and multiple times despite optimal standard therapies. However, early recurrence is not highlighted at all in the definition of “aggressive” tumors. In the definition of “refractory” tumors, tumor recurrence or regrowth within 6 months after surgery is used to distinguish refractory PAs from typical benign adenomas. There is still no definite cutoff value for identifying early tumor recurrence to distinguish refractory PAs from truly benign adenomas, and the cutoff for identifying early recurrence (<6 months postoperatively) is still controversial (41). However, the use of the <6 months postoperatively time for identifying early recurrence provides an important reference for the early recognition and diagnosis of refractory PAs.

In summary, the prevalence of pituitary tumors has recently increased, but the majority of patients are asymptomatic and do not need treatment. Although the revised classification strategy for pituitary tumors is based on the levels of transcription factors (determined by IHC) and is more practical and reasonable than older strategies, there are still some controversies. The PitNET terminology encompasses the unpredictable malignant behavior seen in the small subset of aggressive adenomas. However, it may add new ambiguity to the origin of PAs and unnecessary anxiety and frustration to the majority of patients with benign PAs. The definition of aggressive PAs mainly relies on clinical behavior but lacks objective biomarkers and unified diagnostic criteria. However, the term “refractory” could accurately represent the characteristics of these tumors, including clinical behaviors, radiological features, and pathologic characteristics. Moreover, the diagnostic criteria for refractory PAs are stricter, more objective, and more accurate than those for aggressive PAs.

## Author Contributions

All authors listed have made a substantial, direct, and intellectual contribution to the work, and approved it for publication.

## Funding

Financial support for this study was provided by the Scientific Research Project of Capital Health Development in 2020 (grant number: 2020-2-2058) and the Beijing Natural Science Foundation (grant number: 7172057). The funding institutions had no role in the design of the study, data collection and analysis, the decision to publish, or the preparation of the manuscript.

## Conflict of Interest

The authors declare that the research was conducted in the absence of any commercial or financial relationships that could be construed as a potential conflict of interest.

## References

[1] Chatzellis E Alexandraki KI Androulakis II Kaltsas G. Aggressive Pituitary Tumors. Neuroendocrinology (2015) 101(2):87–104. 10.1159/000371806 25571935

[2] Lopes MBS. The 2017 World Health Organization classification of tumors of the pituitary gland: a summary. Acta Neuropathol (2017) 134(4):521–35. 10.1007/s00401-017-1769-8 28821944

[3] Laws ER Penn DL Repetti CS. Advances and controversies in the classification and grading of pituitary tumors. J Endocrinol Invest (2019) 42(2):129–35. 10.1007/s40618-018-0901-5 29858984

[4] Asa SL Ezzat S. The pathogenesis of pituitary tumours. Nat Rev Cancer (2002) 2(11):836–49. 10.1038/nrc926 12415254

[5] Ezzat S Asa SL Couldwell WT Barr CE Dodge WE Vance ML. The prevalence of pituitary adenomas: a systematic review. Cancer-Am Cancer Soc (2004) 101(3):613–9. 10.1002/cncr.20412 15274075

[6] Fernandez A Karavitaki N Wass JA. Prevalence of pituitary adenomas: a community-based, cross-sectional study in Banbury (Oxfordshire, UK). Clin Endocrinol (Oxf) (2010) 72(3):377–82. 10.1111/j.1365-2265.2009.03667.x 19650784

[7] Daly AF Rixhon M Adam C Dempegioti A Tichomirowa MA Beckers A. High prevalence of pituitary adenomas: a cross-sectional study in the province of Liege, Belgium. J Clin Endocrinol Metab (2006) 91(12):4769–75. 10.1210/jc.2006-1668 16968795

[8] Tjörnstrand A Gunnarsson K Evert M Holmberg E Ragnarsson O Rosén T. The incidence rate of pituitary adenomas in western Sweden for the period 2001–2011. Eur J Endocrinol (2014) 171(4):519–26. 10.1530/EJE-14-0144 25084775

[9] Agustsson TT Baldvinsdottir T Jonasson JG Olafsdottir E Steinthorsdottir V Sigurdsson G. The epidemiology of pituitary adenomas in Iceland, 1955-2012: a nationwide population-based study. Eur J Endocrinol (2015) 173(5):655–64. 10.1530/EJE-15-0189 26423473

[10] Freda PU Beckers AM Katznelson L Molitch ME Montori VM Post KD. Pituitary Incidentaloma: An Endocrine Society Clinical Practice Guideline. J Clin Endocrinol Metab (2011) 96(4):894–904. 10.1210/jc.2010-1048 21474686 PMC5393422

[11] Boguszewski CL de Castro Musolino NR Kasuki L. Management of pituitary incidentaloma. Best Pract Res Cl En (2019) 33(2):101268. 10.1016/j.beem.2019.04.002 31027975

[12] Gsponer J De Tribolet N Deruaz JP Janzer R Uske A Mirimanoff RO. Diagnosis, treatment, and outcome of pituitary tumors and other abnormal intrasellar masses. Retrospective analysis of 353 patients. Med (Baltimore) (1999) 78(4):236–69. 10.1097/00005792-199907000-00004 10424206

[13] Anagnostis P Adamidou F Polyzos SA Efstathiadou Z Panagiotou A Kita M. Pituitary incidentalomas: a single-centre experience. Int J Clin Pract (2011) 65(2):172–7. 10.1111/j.1742-1241.2010.02537.x 21235697

[14] Sanno N Oyama K Tahara S Teramoto A Kato Y. A survey of pituitary incidentaloma in Japan. Eur J Endocrinol (2003) 149(2):123–7. 10.1530/eje.0.1490123 12887289

[15] Ho KKY Fleseriu M Wass J van der Lely A Barkan A Giustina A. A tale of pituitary adenomas: to NET or not to NET. Pituitary (2019) 22(6):569–73. 10.1007/s11102-019-00988-2 31571098

[16] Daly AF Beckers A. The Epidemiology of Pituitary Adenomas. Endocrinol Metab Clin North Am (2020) 49(3):347–55. 10.1016/j.ecl.2020.04.002 32741475

[17] Molitch ME. Diagnosis and Treatment of Pituitary Adenomas: A Review. JAMA (2017) 317(5):516–24. 10.1001/jama.2016.19699 28170483

[18] Heaney AP. Pituitary Carcinoma: Difficult Diagnosis and Treatment. J Clin Endocrinol Metab (2011) 96(12):3649–60. 10.1210/jc.2011-2031 PMC3277423 21956419

[19] Sav A Rotondo F Syro LV Di Ieva A Cusimano MD Kovacs K. Invasive, Atypical and Aggressive Pituitary Adenomas and Carcinomas. Endocr Metab Clin (2015) 44(1):99–104. 10.1016/j.ecl.2014.10.008 25732646

[20] Inoshita N Nishioka H. The 2017 WHO classification of pituitary adenoma: overview and comments. Brain Tumor Pathol (2018) 35(2):51–6. 10.1007/s10014-018-0314-3 29687298

[21] Mete O Lopes MB. Overview of the 2017 WHO Classification of Pituitary Tumors. Endocr Pathol (2017) 28(3):228–43. 10.1007/s12022-017-9498-z 28766057

[22] Raverot G Burman P McCormack A Heaney A Petersenn S Popovic V. European Society of Endocrinology Clinical Practice Guidelines for the management of aggressive pituitary tumours and carcinomas. Eur J Endocrinol (2017) 178(1):G1–G24. 10.1530/EJE-17-0796 29046323

[23] Shibuya M. Welcoming the new WHO classification of pituitary tumors 2017: revolution in TTF-1-positive posterior pituitary tumors. Brain Tumor Pathol (2018) 35(2):62–70. 10.1007/s10014-018-0311-6 29500747

[24] Nishioka H Inoshita N. New WHO classification of pituitary adenomas (4th edition): assessment of pituitary transcription factors and the prognostic histological factors. Brain Tumor Pathol (2018) 35(2):57–61. 10.1007/s10014-017-0307-7 29318396

[25] Asa SL Casar-Borota O Chanson P Delgrange E Earls P Ezzat S. From pituitary adenoma to pituitary neuroendocrine tumor (PitNET): an International Pituitary Pathology Club proposal. Endocr-Relat Cancer (2017) 24(4):C5–8. 10.1530/ERC-17-0004 28264912

[26] Villa C Vasiljevic A Jaffrain-Rea ML Ansorge O Asioli S Barresi V. A standardised diagnostic approach to pituitary neuroendocrine tumours (PitNETs): a European Pituitary Pathology Group (EPPG) proposal. Virchows Arch (2019) 475(6):687–92. 10.1007/s00428-019-02655-0 31578606

[27] Trouillas J Jaffrain-Rea M Vasiljevic A Raverot G Roncaroli F Villa C. How to Classify Pituitary Neuroendocrine Tumors (PitNET)s in 2020. Cancers (2020) 12(2):514. 10.3390/cancers12020514 PMC7072139 32098443

[28] Sathyakumar R Chacko G. Newer Concepts in the Classification of Pituitary Adenomas. Neurol India (2020) 68(Supplement):S7–12. 10.4103/0028-3886.287667 32611886

[29] Kasuki L Raverot G. Definition and diagnosis of aggressive pituitary tumors. Rev Endocr Metab Disord (2019) 21(2):203–8. 10.1007/s11154-019-09531-x 31808044

[30] Dehdashti AR Chakraborty S. Aggressive pituitary adenomas: is pathology the only feature of aggressiveness? Acta Neurochir (2018) 160(1):57–8. 10.1007/s00701-017-3398-3 29177597

[31] Asa SL Ezzat S. Aggressive Pituitary Tumors or Localized Pituitary Carcinomas: Defining Pituitary Tumors. Expert Rev Endocrinol Metab (2016) 11(2):149–62. 10.1586/17446651.2016.1153422 30058871

[32] Trouillas J Burman P McCormack A Petersenn S Popovic V Dekkers O. Aggressive pituitary tumours and carcinomas: two sides of the same coin? Eur J Endocrinol (2018) 178(6):C7–9. 10.1530/EJE-18-0250 29588294

[33] Micko ASG Wöhrer A Wolfsberger S Knosp E. Invasion of the cavernous sinus space in pituitary adenomas: endoscopic verification and its correlation with an MRI-based classification. J Neurosurg (2015) 122(4):803–11. 10.3171/2014.12.JNS141083 25658782

[34] Knosp E Steiner E Kitz K Matula C. Pituitary adenomas with invasion of the cavernous sinus space: a magnetic resonance imaging classification compared with surgical findings. Neurosurgery (1993) 33(4):610–7; discussion 617-8. 10.1227/00006123-199310000-00008 8232800

[35] Dallapiazza R Bond AE Grober Y Louis RG Payne SC Oldfield EH. Retrospective analysis of a concurrent series of microscopic versus endoscopic transsphenoidal surgeries for Knosp Grades 0-2 nonfunctioning pituitary macroadenomas at a single institution. J Neurosurg (2014) 121(3):511–7. 10.3171/2014.6.JNS131321 24995783

[36] Dai C Feng M Liu X Ma S Sun B Bao X. Refractory pituitary adenoma: a novel classification for pituitary tumors. Oncotarget (2016) 7(50):83657–68. 10.18632/oncotarget.13274 PMC5347795 27845901

[37] Dai C Liu X Ma W Wang R. The Treatment of Refractory Pituitary Adenomas. Front Endocrinol (Lausanne) (2019) 10:334. 10.3389/fendo.2019.00334 31191457 PMC6548863

[38] Dworakowska D Grossman AB. Aggressive and malignant pituitary tumours: state-of-the-art. Endocr Relat Cancer (2018) 25(11):R559–75. 10.1530/ERC-18-0228 30306782

[39] Salehi F Agur A Scheithauer BW Kovacs K Lloyd RV Cusimano M. Ki-67 in pituitary neoplasms: a review–part I. Neurosurgery (2009) 65(3):429. 10.1227/01.NEU.0000349930.66434.82 19687686

[40] Ilie MD Jouanneau E Raverot G. Aggressive Pituitary Adenomas and Carcinomas. Endocr Metab Clin (2020) 49(3):505–15. 10.1016/j.ecl.2020.05.008 32741485

[41] Heaney A. Management of aggressive pituitary adenomas and pituitary carcinomas. J Neuro-Oncol (2014) 117(3):459–68. 10.1007/s11060-014-1413-6 24584748

